# Risk Factors and Adverse Perinatal Outcomes among Term and Preterm Infants Born Small-for-Gestational-Age: Secondary Analyses of the WHO Multi-Country Survey on Maternal and Newborn Health

**DOI:** 10.1371/journal.pone.0105155

**Published:** 2014-08-13

**Authors:** Erika Ota, Togoobaatar Ganchimeg, Naho Morisaki, Joshua P. Vogel, Cynthia Pileggi, Eduardo Ortiz-Panozo, João P. Souza, Rintaro Mori, on behalf of the WHO Multi-Country Survey on Maternal and Newborn Health Research Network

**Affiliations:** 1 Department of Health Policy, National Center for Child Health and Development, Tokyo, Japan; 2 Department of Paediatrics, Graduate School of Medicine, University of Tokyo, Tokyo, Japan; 3 UNDP/UNFPA/UNICEF/WHO/World Bank Special Programme of Research, Development and Research Training in Human Reproduction (HRP), Department of Reproductive Health and Research, World Health Organization, Geneva, Switzerland; 4 School of Population Health, University of Western Australia, Perth, Australia; 5 Center for Population Health Research, National Institute of Public Health, Cuernavaca, Morelos, Mexico; VU University Medical Center, Netherlands

## Abstract

**Background:**

Small for gestational age (SGA) is not only a major indicator of perinatal mortality and morbidity, but also the morbidity risks in later in life. We aim to estimate the association between the birth of SGA infants and the risk factors and adverse perinatal outcomes among twenty-nine countries in Africa, Latin America, the Middle East and Asia in 359 health facilities in 2010–11.

**Methods:**

We analysed facility-based, cross-sectional data from the WHO Multi-country Survey on Maternal and Newborn Health. We constructed multilevel logistic regression models with random effects for facilities and countries to estimate the risk factors for SGA infants using country-specific birthweight reference standards in preterm and term delivery, and SGA’s association with adverse perinatal outcomes. We compared the risks and adverse perinatal outcomes with appropriate for gestational age (AGA) infants categorized by preterm and term delivery.

**Results:**

A total of 295,829 singleton infants delivered were analysed. The overall prevalence of SGA was highest in Cambodia (18.8%), Nepal (17.9%), the Occupied Palestinian Territory (16.1%), and Japan (16.0%), while the lowest was observed in Afghanistan (4.8%), Uganda (6.6%) and Thailand (9.7%). The risk of preterm SGA infants was significantly higher among nulliparous mothers and mothers with chronic hypertension and preeclampsia/eclampsia (aOR: 2.89; 95% CI: 2.55–3.28) compared with AGA infants. Higher risks of term SGA were observed among sociodemographic factors and women with preeclampsia/eclampsia, anaemia and other medical conditions. Multiparity (> = 3) (AOR: 0.88; 95% CI: 0.83–0.92) was a protective factor for term SGA. The risk of perinatal mortality was significantly higher in preterm SGA deliveries in low to high HDI countries.

**Conclusion:**

Preterm SGA is associated with medical conditions related to preeclampsia, but not with sociodemographic status. Term SGA is associated with sociodemographic status and various medical conditions.

## Introduction

Small for gestational age (SGA) refers to infants whose size and weight is less than the average range for infants of the same gestational age. GA is not only a major indicator of perinatal mortality and morbidity [1], [2], but also increases the risk of chronic diseases such as cardiovascular disease and diabetes or developmental outcomes later in life [3], [4]. In a UK population-based cohort study from 1997 to 2003, 43% of stillbirths were related to SGA [5]. Among 135 million infants born in low- and middle­income countries (LMICs) in 2010, it is estimated that 29.7 million (22%) were born term-SGA, 10.9 million (8.1%) were born preterm appropriate­for­gestational­age (AGA), and 2.8 million (2.1%) were born preterm-SGA [6]. However, it is a great challenge to define SGA in various ethnic groups in an international comparative study. Based on the secondary analysis using 20 cohort studies for national and regional estimates of SGA babies, 62% of SGA deliveries occurred in India, and 56% occurred in Nepal [7]. This overestimation arose due to the use of the Alexander reference in the analysis, which adapted very high-income country group (US) data from 1991 to low- and middle-income countries. The country-specific birth reference was required to avoid an under- or overestimate of SGA status, especially in low- and middle-income countries. Birthweight references based on neonatal birthweight at each gestational week have been used for nearly 50 years. This type of reference is not so effective under diagnosis in the early gestational weeks, especially for preterm SGA. Therefore, ultrasound-based estimated references of fetal weight are more suitable to overcome this problem. Mikolajczyk developed an ultrasound-based generic global reference to measure fetal weight and birthweight in low-, middle- and high-income settings [8]. Although this country-specific reference has already been used in a previous study to define macrosomia for international comparison [9], our study is the first to use this global reference to define SGA for international comparison.

The cause of SGA is multifactorial, and comprised of maternal, placental, fetal or environmental factors. Identified maternal factors of SGA include demographic variables and medical conditions, such as maternal age [10], [11], nulliparity [11], [12], cigarette smoking [12]–[15], short stature [12], caffeine intake [16], low or high maternal body mass index (BMI) [10], hypertension and preeclampsia [11], [12], [17], psychosocial stress [15], and socioeconomic status, including education [14], [17]–[20]. Conflicting evidence exists for increased [21]–[23] or decreased [24], [25], or unchanged [26], [27] neonatal mortality and morbidity rates for preterm SGA compared with preterm AGA. Risk factors, interventions and sequelae for preterm SGA might differ from term SGA. Despite the high prevalence of SGA, only a limited number of studies exist due to a lack of gestational age data, especially in LMICs. Furthermore, few studies have considered risk factors for SGA in preterm and term deliveries compared with preterm AGA [10], [23], [28]. Therefore, we aimed to explore trends and risk factors associated with SGA and its mortality in preterm and term deliveries across multiple low- to very high- income countries by taking advantage of the WHO Multi-country Survey on Maternal and Newborn Health data, which covers 29 low- to very high-income countries globally.

## Methods

This is a secondary data analysis of the WHO Multi-country Survey on Maternal and Newborn Health, which was conducted in 359 health facilities across 29 countries in Africa, Asia, Latin America and the Middle East. Methodological details of this survey have been published elsewhere [29], [30]. In brief, a multistage cluster sampling method was used to obtain samples of health facilities in two provinces and each capital city of the 29 randomly selected countries. All women admitted for delivery plus all women with severe maternal outcomes regardless of gestational age were recruited in the study. Individual data on demographics and reproductive characteristics, medical conditions during pregnancy, birth outcomes, and complications were collected from the participants’ medical records. Health facility capacity data were obtained, such as the capabilities of essential and comprehensive obstetric and neonatal healthcare services, laboratory tests, and human resources and training. The study was implemented concurrently in 29 countries over two to four months from May 2010 to December 2012.

### Study population and statistical analysis

The study population was restricted to pregnancies of at least 28 gestational weeks for comparability of viable gestational age between countries, and singleton births with no congenital malformation. We excluded deliveries with missing data on birthweight, gestational age, and infant gender, as well as pregnancies that lasted less than 22 weeks or more than 42 weeks with congenital malformation.

To overcome the existing deficiency in birthweight references in LMICs, and taking into account birthweight variations across countries, we adopted methodology to generate local (country-specific) fetal weight and birthweight references developed by Mikolajczyk et al. [8].

To generate a country weight-reference standard, first we used the mean birthweight for infants born to married mothers aged 20–34 years with schooling years ≥12, who had no pregnancy complications, and who vaginally delivered singleton infants with no complications at 40 completed weeks of gestation (40 weeks+0 days to 40 weeks+6 days). Next, we based the birthweight (mean and SD) reference on a gestational age of 40 weeks, and we obtained the mean fetal-weight and percentiles across each gestational week for all countries participating in this study. We defined SGA as a birthweight below the 10^th^ percentile, AGA as between the 10^th^ and 90^th^ percentiles and large-for-gestational age (LGA) as above the 90^th^ percentile at the gestational ages of 28 to 41 weeks by infant gender. The study population was restricted to deliveries with a birthweight below the 90^th^ percentile, excluding LGA due to the condition’s high risk of adverse birth outcomes.

We considered the following variables as exposures at the individual level and further categorized them as shown in tables: maternal age defined as completed years at the time of delivery; marital status; years of education, parity; presence of chronic hypertension, preeclampsia or eclampsia, severe anaemia with haemoglobin <7 mg/dl, malaria or dengue, HIV or AIDS and other conditions defined as the presence of disease or injury affecting the heart, lungs, liver and kidneys. Additionally, we adjusted our analysis for facility capacity and the human development index (HDI). Facility capacity was used in previous studies and is defined as the total score of essential and additional services provided by health facilities with further categorization into high, medium and low capacity [31]. The human development index (HDI) for each country was adopted from 2012 UN development program estimates [32].

Perinatal outcomes considered in the study were fresh stillbirths (excluding macerated stillbirths), early neonatal death, perinatal death (both fresh stillbirth and early neonatal death) and neonatal near miss [33]. Neonatal near miss is defined as a neonate who survived a life-threatening condition and presented with any of the following conditions: any intubation at birth or anytime within the first week of life, nasal continuous positive airway pressure, surfactant administration, cardiopulmonary resuscitation (cardiac massage), any surgery, or use of any vasoactive drug, anticonvulsants, phototherapy in the first 24 hours, steroids to treat refractory hypoglycaemia, or therapeutic intravenous antibiotics. Early neonatal deaths were defined as intra-hospital deaths that occurred on or before the seventh day after delivery.

### Statistical analysis

We divided our sample into two groups by gestational age: preterm (<37 weeks gestational age) and term (37–41 weeks gestational age) deliveries. The characteristics and outcomes of SGA compared to AGA infants in these groups were analysed separately. We compared preterm SGA vs preterm AGA, term SGA vs term AGA.

We performed the Chi-square test by taking into account the clustering and probability-sampling effects of the survey design. Also, after considering the study sampling design and clustering effects (health facility and country) on individual outcomes, we constructed multilevel logistic regression models with random effects for three levels: individual, facility and country. In our analyses of the association between SGA and fresh stillbirths and early neonatal death, we adjusted for maternal age, marital status, education, parity, medical conditions during pregnancy such as chronic hypertension, preeclampsia/eclampsia, severe anaemia, malaria/dengue and HIV/AIDs at the individual level, and capacity of health facilities at the facility level by four categorised HDI groups. The categories comprised as follows: very high HDI countries included Japan, Qatar and Argentina; high HDI countries included Mexico, Lebanon, Peru, Brazil, Ecuador and Sri Lanka; medium HDI countries included Jordan, China, Thailand, Mongolia, the Occupied Palestinian Territory, Paraguay, Philippines, Vietnam, Nicaragua, India and Cambodia, and low HDI countries included Kenya, Pakistan, Angola, Nigeria, Nepal, Uganda, Afghanistan, the Democratic Republic of Congo and Niger. In the ‘overall category’, we adjusted three-level structure random effects regression models to obtain odds ratios (ORs): individual (level 1), facility (level 2) and country (level 3).

Statistical analysis was conducted using Stata/MP version 12.0 (Stata Corp LP, College Station, Texas) and a *P*-value<0.05 was considered statistically significant.

### Ethics committee approval

The HRP Specialist Panel on Epidemiological Research reviewed and approved the study protocol for technical content. This study was approved by the WHO Ethical Review Committee and the relevant ethical clearance mechanisms in all countries. Written consent from individual participants was not required, although patient records was anonymized and de-identified prior to analysis.

## Results

The WHO Multi-country Survey on Maternal and Newborn Health collected a total of 314,623 women’s data from 359 health facilities in 29 countries. Excluded from the analysis were deliveries with missing gestational age and birthweight (5,392), pregnancies that lasted less than 28 weeks or more than 42 weeks (6,191); multiple births (4,579), infants with congenital malformation (2,041) and missing infant gender (255). After the exclusions were made, a total of 295,829 deliveries were retained in the analysis. Table 1 presents the mean birthweight and the prevalence of SGA by each country. The overall prevalence of SGA was highest in Cambodia (18.8%), Nepal (17.9%), the Occupied Palestinian Territory (16.1%), and Japan (16.0%), while the lowest was observed in Afghanistan (4.8%), Uganda (6.6%) and Thailand (9.7%). With further exclusion of LGA infants, the sample size was reduced to 245,77, consisting of 210,047 (85.5%) AGA and 35,726 (14.5%) SGA infants, including 3,827 (26.6%) preterm SGA and 31,932 (13.8%) term SGA, respectively. Table 2 indicates rates of SGA by maternal and neonatal characteristics in preterm and term deliveries. The rates of both preterm and term SGA deliveries were consistently high across HDI groups.

**Table 1:** Birthweight and proportion of SGA by country.

HDI group	Country(by rank)	Total numberof deliveries	Birthweight[mean (SD)]	Small for gestational age (SGA)			
				All [n (%)]	Gestational age (weeks) [n (%)]		
					≤32	33–36	37–41
Very high	Japan	3,391	2975.3 (397.8)	543 (16.0)	2 (9.5)	27 (18.1)	514 (15.9)
	Qatar	3,744	3285.8 (477.2)	453 (12.1)	3 (42.8)	15 (10.5)	435 (12.1)
	Argentina	9,416	3320.7 (516.7)	1,239 (13.2)	40 (50.6)	122 (25.4)	1,077 (12.5)
High	Mexico	12,759	3054.3 (508.3)	1,669 (13.1)	69 (31.9)	234 (25.3)	1,366 (11.8)
	Lebanon	3,826	3175.5 (478.7)	384(10.0)	10 (23.8)	32 (14.3)	342 (9.6)
	Peru	14,450	3310.3 (521.8)	2,120 (14.7)	68 (40.9)	208 (31.2)	1,844 (13.5)
	Brazil	6,729	3161.0 (529.5)	964 (14.3)	39 (33.9)	116 (21.7)	809 (13.3)
	Ecuador	9,810	3070.1 (493.3)	1,335 (13.6)	45 (39.1)	121 (23.9)	1,169 (12.7)
	Sri Lanka	17,530	2925.5 (464.6)	2,249 (12.8)	37 (26.8)	194 (17.8)	2,018 (12.4)
Medium	Jordan	1,066	3119.2 (542.6)	127 (11.9)	5 (27.7)	13 (15.3)	109 (11.3)
	China	12,780	3277.5 (468.4)	1,303 (10.2)	17 (17.5)	78 (12.8)	1,208 (10.0)
	Thailand	8,687	3062.1 (467.8)	841 (9.7)	19 (18.8)	68 (9.1)	754 (9.6)
	Mongolia	7,095	3390.1 (502.0)	711 (10.0)	20 (25.6)	43 (16.9)	648 (9.6)
	OPT	884	3221.3 (502.6)	142 (16.1)	4 (44.4)	15 (27.3)	123 (15.0)
	Paraguay	3,492	3276.6 (535.2)	369 (10.6)	13 (36.1)	41 (18.5)	315 (9.7)
	Philippines	10,120	2923.9 (490.4)	1,520 (15.0)	40 (21.6)	139 (24.0)	1,341 (14.3)
	Vietnam	14,803	3185.3 (430.9)	2,141 (14.5)	15 (29.4)	91 (24.5)	2,035 (14.2)
	Nicaragua	6,231	3043.2 (496.3)	755 (12.1)	27 (26.0)	73 (18.2)	655 (11.4)
	India	30,034	2652.3 (494.3)	3,416 (11.4)	95 (11.6)	366 (14.8)	2,955 (11.1)
	Cambodia	4,525	3001.4 (481.2)	852 (18.8)	12 (11.7)	45 (31.0)	795 (18.6)
Low	Kenya	18,676	3067.3 (537.2)	2,637 (14.1)	99 (20.9)	251 (23.5)	2,287 (13.3)
	Pakistan	12,656	2945.5 (501.2)	1,316 (10.4)	54 (22.5)	173 (17.3)	1,089 (9.6)
	Angola	9,781	3140.8 (515.0)	1,083 (11.1)	19 (7.4)	24 (8.6)	1,050 (11.2)
	Nigeria	11,048	3126.7 (529.5)	1,247 (11.3)	60 (26.5)	110 (13.7)	1,077 (10.8)
	Nepal	10,474	2888.6 (493.7)	1,874 (17.9)	20 (14.0)	130 (27.5)	1,724 (17.5)
	Uganda	8,522	3227.8 (501.5)	566 (6.6)	23 (23.9)	59 (16.3)	484 (6.0)
	Afghanistan	24,932	3174.7 (455.8)	1,187 (4.8)	35 (14.4)	26 (9.8)	1,126 (4.6)
	DRC	7,631	3013.2 (514.1)	1,037 (13.6)	12 (8.4)	62 (8.9)	963 (14.2)
	Niger	10,737	3098.2 (496.2)	1,679 (15.6)	23 (43.4)	36 (38.7)	1,620 (15.3)
All countries		295,829	3067.3 (527.5)	35,759 (12.1)	915 (21.8)	2,912 (18.6)	31,932 (11.6)

Numbers shown are for singleton births with gestational age 28 to 41 completed weeks.

OPT = Occupied Palestinian Territory; DRC = Democratic Republic of Congo.

**Table 2:** Maternal and neonatal characteristics.

	Preterm delivery (≤36 weeks)		p value	Term delivery (≥37 weeks)		p value
	Total deliveries	SGA [n (%)]		Total deliveries	SGA [n (%)]	
All deliveries	14,360	3,827 (26.6)		231,413	31,899(13.8)	
Age						
<20	1,840	530 (28.8)	p<0.05	25,283	4,508 (17.8)	p<0.001
20–34	10,608	2,753 (25.9)		179,550	24,132 (13.4)	
≥35-	1,912	544 (28.4)		26,580	3,292 (12.3)	
Marital status						
Single	1,684	520 (30.8)	p<0.01	24,077	4,122 (17.1)	p<0.001
Married	12,570	3,277 (26.1)		205,625	27,585 (13.4)	
Education, years						
0	1,936	485 (25.0)	0.466	34,276	4,163 (12.1)	p<0.001
1–6	1,948	501 (25.7)		30,242	4,850 (16.0)	
7–9	2,988	776 (25.9)		44,161	6,386 (14.5)	
10–12	4,234	1,176 (27.8)		67,652	9,852 (14.6)	
>12	2,082	584 (28.1)		37,896	4,641 (12.2)	
Parity						
0	6,766	1,889 (27.9)	p<0.05	102,653	16,831 (16.4)	p<0.001
1–2	5,617	1,420 (25.3)		93,762	11,354 (12.1)	
≥3	1,958	511 (26.1)		34,696	3,681 (10.6)	
Mode of delivery						
Vaginal	8,801	2,109 (23.9)	p<0.001	169,114	23,157 (13.7)	0.298
Caesarean	5,538	1,708 (30.8)		62,022	8,709 (14.0)	
Medical conditions						
Chronic hypertension	228	96 (42.1)	p<0.001	703	134 (19.1)	p<0.001
Pre-/eclampsia	1,680	781 (46.5)	p<0.001	4,207	1,031 (24.5)	p<0.001
Anaemia (HB<7 mg/dl)	608	210 (34.5)	p<0.01	2,791	512 (18.3)	p<0.001
Malaria/dengue	55	17 (30.9)	0.540	193	46 (23.8)	p<0.001
HIV/AIDs	99	26 (26.3)	0.935	845	161 (19.1)	p<0.001
Others	318	115 (36.2)	p<0.01	1,221	218 (17.8)	p<0.001
Infant gender						
Male	7,605	1,994 (26.2)	0.248	118,483	15,856 (13.4)	p<0.001
Female	6,755	1,833 (27.1)		112,930	16,043 (14.2)	
Apgar score at5 minutes <7	1,253	502 (40.1)	p<0.001	4,503	1051 (23.3)	p<0.001
Country HDI						
Very high	768	209 (27.2)	p<0.001	13,725	1,993 (14.5)	p<0.05
High	3,736	1,173 (31.4)		51,470	7,548 (14.7)	
Medium	5,443	1,239 (22.8)		76,203	10,938 (14.4)	
Low	4,13	1,206 (27.3)		90,015	11,420 (12.7)	
Facility capacity						
High	5,053	1,390 (27.5)	0.245	60,857	8,435 (13.8)	0.119
Medium	5,349	1,470 (27.5)		92,575	11,970 (12.9)	
Low	2,552	596 (23.4)		48,288	7,274 (15.1)	

Other medical conditions were included, such as chronic or acute injury or disorders affecting the heart, lungs, liver and kidneys (including pyelonephritis).

Chi-square *p*-values adjusted for survey design.

Table 3 shows risk factors for SGA in preterm and term deliveries. The risk factors of delivering preterm SGA infants were significantly higher compared to AGA risk factors among nulliparous women (adjusted odds ratio [AOR]: 1.17; 95% CI: 1.06–1.29), and women with chronic hypertension (AOR: 1.68; 95% CI: 1.22–2.30) and preeclampsia/eclampsia (AOR: 2.89; 95% CI: 2.55–3.28). Higher risks of term SGA compared with term AGA were observed among younger (AOR: 1.09; 95% CI: 1.04–1.14) and older women (AOR: 1.07; 95% CI: 1.02–1.13), single women (AOR: 1.11; 95% CI: 1.06–1.17), women with 1–6 years of education (AOR: 1.55; 95% CI: 1.46–1.65), nulliparous women (AOR: 1.45; 95% CI: 1.41–1.50), and women with preeclampsia/eclampsia (AOR: 2.05; 95% CI: 1.88–2.23), anaemia (HB<7 mg/dl) (AOR: 1.30; 95% CI: 1.15–1.47), HIV/AIDS (AOR: 1.48; 95% CI: 1.22–1.80), and other medical conditions (AOR: 1.47; 95% CI: 1.24–1.74). Multiparity (> = 3) (AOR: 0.88; 95% CI: 0.83–0.92) was a protective factor for term SGA and, after adjusting for variables, country HDI had no significant association.

**Table 3:** Risk factors for SGA.

	Preterm delivery (≤36 weeks)			Term delivery (≥37 weeks)		
	OR	AOR	95% CI	OR	AOR	95% CI
Age						
<20	1.15*	1.04	(0.89–1.20)	1.39***	1.09	(1.04–1.14)***
20–34	reference					
≥35-	1.13*	1.08	(0.94–1.24)	0.91**	1.07	(1.02–1.13)**
Marital status						
Single	1.27**	1.15	(0.98–1.34)	1.33***	1.11	(1.06–1.17)***
Married	reference					
Education, years						
0	0.85	1.07	(0.88–1.31)	0.99	1.50	(1.41–1.61)***
1–6	0.88	1.03	(0.86–1.23)	1.37***	1.55	(1.46–1.65)***
7–9	0.89	1.01	(0.86–1.19)	1.21***	1.34	(1.27–1.41)***
10–12	0.98	1.02	(0.89–1.18)	1.22***	1.22	(1.17–1.28)***
>12	reference					
Parity						
0	1.14**	1.17	(1.06–1.29)**	1.42***	1.45	(1.41–1.50)***
1–2	reference					
≥3	1.04	0.96	(0.83–1.12)	0.86***	0.88	(0.83–0.92)***
Medical conditions						
Chronic hypertension	2.02***	1.68	(1.22–2.30)**	1.47***	1.20	(0.96–1.49)
Preeclampsia/eclampsia	2.75***	2.89	(2.55–3.28)***	2.06***	2.05	(1.88–2.23)***
Anaemia (HB<7 mg/dl)	1.48***	1.24	(0.99–1.56)	1.41***	1.30	(1.15–1.47)***
Malaria/dengue	1.23	1.16	(0.58–2.32)	1.96***	1.26	(0.83–1.92)
HIV/AIDs	0.98	0.85	(0.50–1.44)	1.47***	1.48	(1.22–1.80)***
Others medicalconditions	1.58**	1.24	(0.92–1.67)	1.36**	1.47	(1.24–1.74)***
Country HDI						
Very high	reference					
High	1.22	1.23	(0.64–2.34)	1.01	0.79	(0.39–1.59)
Medium	0.78*	0.88	(0.47–1.63)	0.98	0.85	(0.51–1.44)
Low	1.01	1.28	(0.68–2.42)	0.85*	0.61	(0.37–1.02)

Other medical conditions were included, such as chronic or acute injury or disorders affecting the heart, lungs, liver and kidneys (including pyelonephritis).

SGA = small-for-gestational age; HDI = Human Development Index; OR = odds ratio; AOR = adjusted odds ratio. Three-level structure random effects regression models were used to obtain ORs: individual (level 1), facility (level 2) and country (level 3).

***p<0.001 **p<0.01 *p<0.05.

Prevalence of adverse perinatal outcomes for SGA by gestational weeks in each HDI country group is presented in Table 4. We observed a significant trend of higher mortality rates in SGA and all deliveries for lower HDI countries (*P*<0.001).

**Table 4:** Prevalence of fresh stillbirths and early neonatal mortality by HDI country groups.

Outcome	SGA [n/N (%)]	HDI country group [n/N (%)]				*p*-value
		Very High	High	Medium	Low	
**Fresh stillbirth**						
All deliveries	2458/244382 (1.0)	31/14426 (0.2)	183/55096 (0.3)	578/81251 (0.7)	1666/93578 (1.8)	p<0.001
SGA deliveries						
≤32	144/797 (18.1)	3/44 (6.8)	20/248(8.1)	41/243 (16.9)	80/262 (30.5)	p<0.001
33–36	169/2748 (6.2)	3/160 (1.9)	27/890 (3.0)	55/920 (6.0)	84/778 (10.8)	p<0.001
≥37	520/31585 (1.7)	3/1987 (0.2)	31/7529 (0.4)	133/10837 (1.2)	353/11232 (3.1)	p<0.001
**Neonatal near miss**						
All live deliveries	11436/228831 (4.8)	454/14417 (3.2)	3210/54736 (5.9)	4550/80108 (5.7)	3222/91006 (3.5)	p<0.001
SGA deliveries						
≤32	355/484 (73.4)	32/40 (80.0)	160/201 (79.6)	115/145 (79.3)	48/98 (49.0)	p = 0.003
33–36	1011/2419 (41.8)	49/155 (31.6)	396/837 (47.3)	358/801 (44.7)	208/626 (33.2)	p = 0.019
≥37	1889/30785 (6.1)	58/1982 (2.9)	441/7480 (5.9)	826/10603 (7.8)	564/10720 (5.3)	p = 0.016
**Early neonatal death**						
All live deliveries	1534/241924 (0.6)	19/14426 (0.1)	160/54913 (0.3)	514/80673 (0.6)	841/91912 (0.9)	p<0.001
SGA deliveries						
≤32	162/653 (24.8)	1/41 (2.4)	23/228 (10.1)	56/202 (27.7)	82/182 (45.1)	p<0.001
33–36	152/2579 (5.9)	2/157(1.3)	23/863 (2.7)	61/865 (7.1)	66/694 (9.5)	p<0.001
≥37	267/31065 (0.9)	3/1984 (0.2)	15/7498 (0.2)	93/10704 (0.9)	156/10879 (1.4)	p<0.001
**Perinatal death**						
All deliveries	3992/244382 (1.6)	50/14457 (0.4)	343/55096 (0.6)	1092/81251 (1.3)	2507/93578 (2.7)	p<0.001
SGA deliveries						
≤32	306/797 (38.4)	4/44 (9.9)	43/248 (17.3)	97/243 (39.9)	162/262 (61.8)	p<0.001
33–36	321/2748 (11.7)	5/160 (3.1)	50/890 (5.6)	116/920 (12.6)	150/778 (19.3)	p<0.001
≥37	787/31585 (2.5)	6/1987 (0.3)	46/7529 (0.6)	226/10837 (2.1)	509/11232 (4.5)	p<0.001

SGA = small-for-gestational age; HDI = Human Development Index Chi-square *p*-values adjusted for survey design.

The association between SGA deliveries and fresh stillbirths, neonatal near miss, early neonatal deaths, and perinatal deaths compared with AGA deliveries by HDI country group are presented in Table 5 and are stratified by preterm and term delivery. For preterm and term SGA, very high HDI countries had no significant increase in fresh stillbirth, early neonatal mortality and perinatal mortality, although low to high HDI countries had risks two to four times higher than preterm AGA. For neonatal near miss, both preterm and term SGA deliveries had 1.7 to 2.7 times significantly higher risk than AGA, although preterm SGA had a higher prevalence of near miss (50% to 80% among neonates of less than 32 weeks’ gestation) than term SGA, irrespective of HDI countries.

**Table 5:** The association between SGA and perinatal outcomes compared with AGA by HDI country groups.

HDI group	Preterm delivery (≤36 weeks)		Term delivery (≥37 weeks)		All deliveries	
	AOR	95% CI	AOR	95% CI	AOR	95% CI
**Fresh stillbirth**						
Very high	0.31	(0.06–1.76)	1.79	(0.29–10.9)	1.46	(0.47–4.51
High	2.31	(1.36–3.93)**	3.00	(1.75–5.12)***	3.70	(2.56–5.33)***
Medium	2.18	(1.62–2.96)***	3.08	(2.43–3.89)***	2.97	(2.47–3.56)***
Low	1.99	(1.54–2.57)***	2.89	(2.47–3.37)***	3.07	(2.69–3.51)***
Overall§	2.01	(1.66–2.42)***	2.95	(2.60–3.36)***	3.07	(2.77–3.41)***
**Neonatal near miss**						
Very high	2.34	(1.47–3.71)***	1.65	(1.13–2.42)**	2.61	(2.02–3.37)***
High	2.60	(2.17–3.11)***	1.69	(1.48–1.93)***	2.47	(2.24–2.71)***
Medium	2.32	(1.98–2.74)***	2.38	(2.17–2.61)***	2.43	(2.26–2.63)***
Low	2.43	(1.97–2.99)***	1.75	(1.57–1.95)***	2.03	(1.85–2.23)***
Overall§	2.65	(2.37–2.96)***	1.99	(1.87–2.12)**	2.39	(2.27–2.51)***
**Early neonatal death**						
Very high	1.19	(0.25–5.74)	1.39	(0.15–12.32)	2.14	(0.67–6.94)
High	3.77	(1.97–6.47)***	2.14	(1.09–4.20)*	3.92	(2.57–5.97)***
Medium	2.77	(2.08–3.68)***	3.44	(2.61–4.56)***	3.56	(2.93–4.32)***
Low	2.92	(2.21–3.83)***	2.94	(2.37–3.63)***	3.53	(3.00–4.16)***
Overall§	2.86	(2.36–3.46)***	3.01	(2.56–3.56)***	3.52	(3.12–3.96)***
**Perinatal death**						
Very high	0.69	(0.22–2.16)	1.78	(0.46–6.82)	1.76	(0.78–3.99)
High	2.89	(1.94–4.31)***	2.63	(1.73–3.99)***	3.80	(2.88–5.02)***
Medium	2.61	(2.10–3.25)***	3.27	(2.72–3.92)***	3.29	(2.88–7.77)***
Low	2.51	(2.06–3.06)***	2.92	(2.58–3.32)***	3.31	(2.98–3.67)***
Overall§	2.50	(2.17–2.87)***	3.00	(2.71–3.32)***	3.31	(3.06–3.59)***

The reference category is infants with a birthweight that is appropriate for gestational age in each subgroup analysis.

SGA = small-for-gestational age; AGA = appropriate-for-gestational age; HDI = Human Development Index, AOR = adjusted odds ratio.

Two-level structure random effects regression models were used to obtain ORs: individual (level 1) and facility (level 2). Adjusted for maternal age, marital status, education, parity, medical conditions during pregnancy such as chronic hypertension, preeclampsia/eclampsia, severe anaemia, malaria/dengue, HIV/AIDs at the individual level, and capacity of health facilities at the facility level.

<?ENTCHAR sect?> Three-level structure random effects regression models were used to obtain ORs: individual (level 1), facility (level 2) and country (level 3). Same adjustment at individual and facility level and additional adjustment for country HDI at the country level.

***p<0.001 **p<0.01 *p<0.05.

## Discussion

### Main findings

We determined the maternal risk factors and adverse perinatal outcomes in preterm- and term-SGA infants in 29 countries globally using a large multi-country dataset. After adjusting for country-, facility- and individual-level effects, we found no association between increased risks of preterm SGA and sociodemographic status, such as age or education, compared with preterm AGA; however, we did observe that nulliparity and medical conditions, such as chronic hypertension and preeclampsia/eclampsia, were significantly associated with increased risks of preterm SGA compared with preterm AGA.

### Strengths and limitations

To the best of our knowledge, this is the most current and extensive multi-country study to compare and examine risk factors and their adverse outcomes in preterm SGA and term SGA deliveries compared with preterm and term AGA deliveries using country-specific generic references. We used SGA criteria that incorporates country-specific reference standards developed by Mikolajczyk et al. [8]. This generic, global reference for fetal-weight and birthweight percentiles is more effective in predicting adverse perinatal outcomes compared with non-customised fetal-weight references, and is easier to use than the customised fetal-weight reference. A large sample size and the use of standardized questionnaires across countries allowed us to examine outcomes and stratify countries by five HDI groups.

Our study has several limitations. First, the quality of the data, especially birthweight and gestational age, is questionable in some countries. Errors might occur in dating the pregnancy, especially in countries where gestational age is based on the last menstrual period or where the birthweight is rounded up or down by a full 100 g. Due to this limitation, we focused on identifying the risk factors of SGA rather than focusing on SGA prevalence in each country.

Another limitation is a lack of data on maternal characteristics that have been noted in previous studies to be associated with the delivery of SGA infants, including smoking, alcohol and caffeine intake, maternal BMI, malnutrition, gestational weight gain, maternal stature, psychosocial stress, interpregnancy interval, and previous history of miscarriage [10]–[16]. Lack of adjustment for these variables may have led to an overestimation of the risk of SGA delivery, especially for women of a younger or older age, with less education or in low HDI-scoring countries.

Lastly, by using multilevel multiple regression analysis we were able to generalize our findings among facility-based settings; however, adverse perinatal outcomes and maternal medical conditions may have been overestimated because only the most severe cases are presented in higher-level facilities. Furthermore, the risk of neonatal mortality and morbidity could be underestimated due to the 7-day period in this study for neonatal follow-up. It should be noted that mortality due to infections, necrotising enterocolitis and other complications may occur after this period. Thus, the outcomes and conditions cannot be considered representative of the general population.

### Interpretation

Our results suggest that nulliparity, chronic hypertension and preeclampsia/eclampsia are associated with a higher risk of preterm SGA. This result is consistent with other studies [18], [34]. In a national birth cohort study in Denmark, Catov et al. found that risk of preterm SGA increased 5.5 (95% confidence interval [CI] 3.2–9.4) times and term SGA increased 1.5 (95% CI 1.0–2.2) times among women with chronic hypertension [34]. The result is also consistent with the findings of Villar et al. who analysed data from WHO antenatal care trials and observed that nulliparity, chronic hypertension and obesity are also risk factors for preeclampsia in developing countries, but not low socioeconomic status [18]. Preeclampsia may cause an inadequate vascular response to abnormal placentation in pregnancy and may represent a distinct pathogenesis, which might affect fetal growth [6], [35]. Increased risk screening in antenatal care visits and referral to higher facilities for high-risk cases at an earlier stage in the pregnancy may help to reduce the incidence of severe preeclampsia or eclampsia.

We found that sociodemographic factors such as age, marital status and education were not significantly associated with the risk of preterm SGA, but sociodemographic status factors were related to term SGA. The results indicated that preterm SGA deliveries are more likely to be related to a maternal medical condition, especially preeclampsia, which tends to terminate the pregnancy earlier. On the other hand, term SGA may be more significantly relevant to lifestyle factors, such as sociodemographic status, malnutrition or other factors, and various medical conditions such as anaemia, HIV/AIDS and others. Our results are consistent with other studies that have observed a significant increased risk of term SGA associated with maternal age [10], [11] and nulliparity [11], [12]. Previous studies confirm that sociodemographic status is associated with a greater risk of SGA, although these studies did not divide SGA by preterm and term delivery [15], [36]. Berg et al. conducted path analysis to examine the relationship between maternal education and SGA using population-based cohort study data and showed that a significantly increased risk of SGA delivery among women with less education was related foremost to maternal smoking and, to some degree, to maternal height [15]. A population-based case-control study using Finnish birth register data also confirmed that between high and low socioeconomic status groups, 50% of the difference in risk of SGA was due to smoking [36].

Very high HDI countries showed no significant increase in the mortality risk for preterm and term SGA deliveries. This might be explained by the high quality of intrapartum care including access to care, human resources and drugs or medical equipment in very high HDI countries, which could reduce the mortality risk for preterm and term SGA deliveries. However, low to high HDI countries had risks two to four times higher compared to preterm AGA. These results are consistent with the population-based secondary analysis conducted in 20 cohorts in LMICs by the Child Health Epidemiology Reference Group (CHERG), which showed that the risk of early neonatal mortality increased about 16 times for preterm SGA delivery compared with preterm non-SGA delivery [37]. The reason for these different degrees of mortality risk might be due to the definition of SGA used by the authors, which they adapted from the US population birthweight reference standard and applied to LMICs. Another population-based cohort study in France showed that the risk of stillbirth was 2.6 times higher in preterm SGA deliveries, which is a similar result to our overall mortality risks [38]. Simchen et al. found that singleton preterm SGA infants had a significantly higher mortality rate with more culture-proven sepsis episodes [23].

In our findings, the risk of mortality in both preterm and term SGA deliveries was higher compared to preterm and term AGA, respectively, in low to high HDI countries. However, very high HDI countries had no significant mortality difference between preterm SGA and AGA, but had higher risks of mortality for term SGA, especially in fresh stillbirths.

Our findings indicate that if LMICs give appropriate care comparable with very high HDI countries, such as including regular risk screening in antenatal care visits and providing adequate treatment and care to those who need treatment at an earlier stage, it might be possible to decrease perinatal mortality among preterm SGA infants. Term SGA infants were three to four times significantly more likely to experience perinatal mortality than term AGA infants, irrespective of HDI groups. This finding supports Lubchenco’s report from 1976, which found that the risk of neonatal mortality was six times more likely in term SGA infants compared with term AGA infants [39]. Risk of perinatal mortality is significantly higher among term SGA deliveries compared with preterm AGA deliveries, irrespective of quality of care.

Neonatal near miss is higher risk, irrespective of HDI, although it has a high prevalence in neonates born at less than 32 weeks’ gestation. In very high HDI countries, 80% of neonates born at less than 32 gestational weeks experienced neonatal near miss, although perinatal mortality was around 11%. In low HDI countries, 49% of neonates born at less than 32 gestational weeks experienced neonatal near miss, and 70% of them died. The quality of neonatal intensive care is vital to prevent mortality.

Neonatal clinical management should be considered in the development of health policies for reducing neonatal mortality, such as screening high-risk neonates for early complications and the referral of pregnant women with hypertensive diseases for delivery in health facilities with special care units. Careful follow-up is necessary for SGA neonates who are at a higher risk of acquiring non-communicable diseases in the future.

Further research could define SGA using the customized rather than standard intrauterine growth curves, especially for countries that adopt curves based on populations from diverse ethnic groups. Ideally the standard questionnaire should include variables such as weight gain during pregnancy and pre-pregnancy BMI.

## Conclusion

Our results demonstrate that preterm SGA is associated with medical conditions related to chronic hypertension and preeclampsia/eclampsia, but is not associated with sociodemographic status. This result clearly identified that global prevention for preterm SGA should mainly focus on preeclampsia. Term SGA is associated with sociodemographic status and various medical conditions. Risk of fresh stillbirth and neonatal death was two to three times higher in preterm SGA in LMICs, except in the very high HDI group. Term SGA was significantly associated with perinatal deaths irrespective of HDI categories.

## References

[1] Lee AC, Katz J, Blencowe H, Cousens S, Kozuki N, et al (2013) National and regional estimates of term and preterm babies born small for gestational age in 138 low-income and middle-income countries in 2010. The Lancet Global Health 1: e26–e36. 25103583 10.1016/S2214-109X(13)70006-8 PMC4221634

[2] Qiu X, Lodha A, Shah PS, Sankaran K, Seshia MM, et al (2012) Neonatal outcomes of small for gestational age preterm infants in Canada. Am J Perinatol 29: 87–94. 22131047 10.1055/s-0031-1295647

[3] Risnes KR, Vatten LJ, Baker JL, Jameson K, Sovio U, et al (2011) Birthweight and mortality in adulthood: a systematic review and meta-analysis. Int J Epidemiol 40: 647–661. 21324938 10.1093/ije/dyq267

[4] Larroque B, Bertrais S, Czernichow P, Leger J (2001) School difficulties in 20-year-olds who were born small for gestational age at term in a regional cohort study. Pediatrics 108: 111–115. 11433062 10.1542/peds.108.1.111

[5] Gardosi J, Kady SM, McGeown P, Francis A, Tonks A (2005) Classification of stillbirth by relevant condition at death (ReCoDe): population based cohort study. BMJ 331: 1113–1117. 16236774 10.1136/bmj.38629.587639.7C PMC1283273

[6] Sheppard BL, Bonnar J (1999) Uteroplacental hemostasis in intrauterine fetal growth retardation. Semin Thromb Hemost 25: 443–446. 10625199 10.1055/s-2007-994947

[7] Katz J, Lee AC, Kozuki N, Lawn JE, Cousens S, et al (2013) Mortality risk in preterm and small-for-gestational-age infants in low-income and middle-income countries: a pooled country analysis. The Lancet 382: 417–425. 10.1016/S0140-6736(13)60993-9 PMC3796350 23746775

[8] Mikolajczyk RT, Zhang J, Betran AP, Souza JP, Mori R, et al (2011) A global reference for fetal-weight and birthweight percentiles. Lancet 377: 1855–1861. 21621717 10.1016/S0140-6736(11)60364-4

[9] Koyanagi A, Zhang J, Dagvadorj A, Hirayama F, Shibuya K, et al (2013) Macrosomia in 23 developing countries: an analysis of a multicountry, facility-based, cross-sectional survey. Lancet 381: 476–483. 23290494 10.1016/S0140-6736(12)61605-5

[10] Zeitlin JA, Ancel PY, Saurel-Cubizolles MJ, Papiernik E (2001) Are risk factors the same for small for gestational age versus other preterm births? Am J Obstet Gynecol 185: 208–215. 11483930 10.1067/mob.2001.114869

[11] Anderson NH, Sadler LC, Stewart AW, Fyfe EM, McCowan LM (2013) Independent risk factors for infants who are small for gestational age by customised birthweight centiles in a multi-ethnic New Zealand population. Aust N Z J Obstet Gynaecol 53: 136–142. 23130970 10.1111/ajo.12016

[12] Thompson JM, Clark PM, Robinson E, Becroft DM, Pattison NS, et al (2001) Risk factors for small-for-gestational-age babies: The Auckland Birthweight Collaborative Study. J Paediatr Child Health 37: 369–375. 11532057 10.1046/j.1440-1754.2001.00684.x

[13] Gissler M, Merilainen J, Vuori E, Hemminki E (2003) Register based monitoring shows decreasing socioeconomic differences in Finnish perinatal health. J Epidemiol Community Health 57: 433–439. 12775790 10.1136/jech.57.6.433 PMC1732478

[14] Erickson AC, Arbour LT (2012) Heavy smoking during pregnancy as a marker for other risk factors of adverse birth outcomes: a population-based study in British Columbia, Canada. BMC Public Health 12: 102. 22304990 10.1186/1471-2458-12-102 PMC3339515

[15] Paarlberg KM, Vingerhoets AJ, Passchier J, Dekker GA, Heinen AG, et al (1999) Psychosocial predictors of low birthweight: a prospective study. Br J Obstet Gynaecol 106: 834–841. 10453835 10.1111/j.1471-0528.1999.tb08406.x

[16] CARE (2008) Maternal caffeine intake during pregnancy and risk of fetal growth restriction: a large prospective observational study. BMJ 337: a2332. 18981029 10.1136/bmj.a2332 PMC2577203

[17] Clausson B, Cnattingius S, Axelsson O (1998) Preterm and term births of small for gestational age infants: a population-based study of risk factors among nulliparous women. Br J Obstet Gynaecol 105: 1011–1017. 9763054 10.1111/j.1471-0528.1998.tb10266.x

[18] Fairley L, Leyland AH (2006) Social class inequalities in perinatal outcomes: Scotland 1980–2000. J Epidemiol Community Health 60: 31–36. 16361452 10.1136/jech.2005.038380 PMC2465545

[19] Raum E, Arabin B, Schlaud M, Walter U, Schwartz FW (2001) The impact of maternal education on intrauterine growth: a comparison of former West and East Germany. Int J Epidemiol 30: 81–87. 11171862 10.1093/ije/30.1.81

[20] Kramer MS, Seguin L, Lydon J, Goulet L (2000) Socio-economic disparities in pregnancy outcome: why do the poor fare so poorly? Paediatr Perinat Epidemiol 14: 194–210. 10949211 10.1046/j.1365-3016.2000.00266.x

[21] Tyson JE, Kennedy K, Broyles S, Rosenfeld CR (1995) The small for gestational age infant: accelerated or delayed pulmonary maturation? Increased or decreased survival? Pediatrics 95: 534–538. 7700754

[22] Piper JM, Xenakis EM, McFarland M, Elliott BD, Berkus MD, et al (1996) Do growth-retarded premature infants have different rates of perinatal morbidity and mortality than appropriately grown premature infants? Obstet Gynecol 87: 169–174. 8559517 10.1016/0029-7844(95)00400-9

[23] Simchen MJ, Beiner ME, Strauss-Liviathan N, Dulitzky M, Kuint J, et al (2000) Neonatal outcome in growth-restricted versus appropriately grown preterm infants. Am J Perinatol 17: 187–192. 11041440 10.1055/s-2000-9423

[24] Procianoy RS, Garcia-Prats JA, Adams JM, Silvers A, Rudolph AJ (1980) Hyaline membrane disease and intraventricular haemorrhage in small for gestational age infants. Arch Dis Child 55: 502–505. 7436499 10.1136/adc.55.7.502 PMC1626792

[25] Laatikainen TJ, Raisanen IJ, Salminen KR (1988) Corticotropin-releasing hormone in amniotic fluid during gestation and labor and in relation to fetal lung maturation. Am J Obstet Gynecol 159: 891–895. 3263045 10.1016/s0002-9378(88)80163-7

[26] Friedman SA, Schiff E, Kao L, Sibai BM (1995) Neonatal outcome after preterm delivery for preeclampsia. Am J Obstet Gynecol 172: 1785–1788 discussion 1788–1792. 7778633 10.1016/0002-9378(95)91412-9

[27] Schiff E, Friedman SA, Mercer BM, Sibai BM (1993) Fetal lung maturity is not accelerated in preeclamptic pregnancies. Am J Obstet Gynecol 169: 1096–1101. 8238166 10.1016/0002-9378(93)90262-h

[28] Heinonen K, Matilainen R, Koski H, Launiala K (1985) Intrauterine growth retardation (IUGR) in pre-term infants. J Perinat Med 13: 171–178. 4057033 10.1515/jpme.1985.13.4.171

[29] Stotts AL, Schmitz JM, Grabowski J (2003) Concurrent treatment for alcohol and tobacco dependence: are patients ready to quit both? Drug Alcohol Depend 69: 1–7. 12536060 10.1016/s0376-8716(02)00227-2

[30] Souza JP, Gulmezoglu AM, Vogel J, Carroli G, Lumbiganon P, et al (2013) Moving beyond essential interventions for reduction of maternal mortality (the WHO Multicountry Survey on Maternal and Newborn Health): a cross-sectional study. Lancet 381: 1747–1755. 23683641 10.1016/S0140-6736(13)60686-8

[31] Joshua PV, Souza JP, Gulmezoglu AM, Mori R, Morisaki N, et al. (2013 (In press)) Maternal complications and perinatal mortality: findings of the World Health Organization Multi-country Survey on Maternal and Newborn Health. BJOG. 10.1111/1471-0528.12633 24641538

[32] Malik K (2013) Human Development Report 2013. United Nations Development Programme, New York.

[33] Pileggi C, Camelo JSJ, Perdoná GC, Mussi-Pinhata MM, Cecatti JG, et al. (2013 (In press)) Development of criteria for identifying neonatal near miss cases: analysis of two WHO multi-country cross sectional studies. BJOG. 10.1111/1471-0528.12637 24641541

[34] Catov JM, Nohr EA, Olsen J, Ness RB (2008) Chronic hypertension related to risk for preterm and term small for gestational age births. Obstet Gynecol 112: 290–296. 18669725 10.1097/AOG.0b013e31817f589b PMC2596352

[35] Kaufmann P, Black S, Huppertz B (2003) Endovascular trophoblast invasion: implications for the pathogenesis of intrauterine growth retardation and preeclampsia. Biol Reprod 69: 1–7. 12620937 10.1095/biolreprod.102.014977

[36] Raisanen S, Gissler M, Sankilampi U, Saari J, Kramer MR, et al (2013) Contribution of socioeconomic status to the risk of small for gestational age infants–a population-based study of 1,390,165 singleton live births in Finland. Int J Equity Health 12: 28. 23634813 10.1186/1475-9276-12-28 PMC3651324

[37] Katz J, Lee AC, Kozuki N, Lawn JE, Cousens S, et al. (2013) Mortality risk in preterm and small-for-gestational-age infants in low-income and middle-income countries: a pooled country analysis. Lancet. 10.1016/S0140-6736(13)60993-9 PMC3796350 23746775

[38] Ferdynus C, Quantin C, Abrahamowicz M, Burguet A, Sagot P, et al. (2013) Comparison of the ability of alternative birthweight and fetal weight standards to identify preterm newborns at increased risk of perinatal death. BJOG. 10.1111/1471-0528.12282 23721356

[39] Lubchenco L (1976) The high risk infant: interauterine growth and neonetal morbidity and mortality. Philadelphia: WB Saunders.

